# Methodological choices in brucellosis burden of disease assessments: A systematic review

**DOI:** 10.1371/journal.pntd.0010468

**Published:** 2022-12-13

**Authors:** Carlotta Di Bari, Narmada Venkateswaran, Mieghan Bruce, Christina Fastl, Ben Huntington, Grace T. Patterson, Jonathan Rushton, Paul Torgerson, David M. Pigott, Brecht Devleesschauwer

**Affiliations:** 1 GBADs programme -, Liverpool, United Kingdom; 2 Department of Epidemiology and Public Health, Sciensano, Brussels, Belgium; 3 Department of Health Metrics Sciences, Institute for Health Metrics and Evaluation, University of Washington, Seattle, Washington, United States of America; 4 Centre for Biosecurity and One Health, School of Veterinary Medicine, Harry Butler Institute, Murdoch University, Perth, Western Australia; 5 Institute of Infection, Veterinary and Ecological Sciences, University of Liverpool, Liverpool, United Kingdom; 6 Pengwern Animal Health Ltd, Wallasey Wirral, Merseyside, United Kingdom; 7 Department of Population Medicine, Ontario Veterinary College, University of Guelph, Guelph, Ontario, Canada; 8 Section of Epidemiology, Vetsuisse Faculty, University of Zurich, Zurich, Switzerland; 9 Department of Translational Physiology, Infectiology and Public Health, Ghent University, Merelbeke, Belgium; Babol University of Medical Science, ISLAMIC REPUBLIC OF IRAN

## Abstract

**Background:**

Foodborne and zoonotic diseases such as brucellosis present many challenges to public health and economic welfare. Increasingly, researchers and public health institutes use disability-adjusted life years (DALYs) to generate a comprehensive comparison of the population health impact of these conditions. DALYs calculations, however, entail a number of methodological choices and assumptions, with data gaps and uncertainties to accommodate. Thisreview identifies existing brucellosis burden of disease studies and analyzes their methodological choices, assumptions, and uncertainties. It supports the Global Burden of Animal Diseases programme in the development of a systematic methodology to describe the impact of animal diseases on society, including human health.

**Methods/Principal findings:**

A systematic search for brucellosis burden of disease calculations was conducted in pre-selected international and grey literature databases. Using a standardized reporting framework, we evaluated each estimate on a variety of key methodological assumptions necessary to compute a DALY. Fourteen studies satisfied the inclusion criteria (human brucellosis and quantification of DALYs). One study reported estimates at the global level, the rest were national or subnational assessments. Data regarding different methodological choices were extracted, including detailed assessments of the adopted disease models. Most studies retrieved brucellosis epidemiological data from administrative registries. Incidence data were often estimated on the basis of laboratory-confirmed tests. Not all studies included mortality estimates (Years of Life Lost) in their assessments due to lack of data or the assumption that brucellosis is not a fatal disease. Only two studies used a model with variable health states and corresponding disability weights. The rest used a simplified singular health state approach. Wide variation was seen in the duration chosen for brucellosis, ranging from 2 weeks to 4.5 years, irrespective of the whether a chronic state was included.

**Conclusion:**

Available brucellosis burden of disease assessments vary widely in their methodology and assumptions. Further research is needed to better characterize the clinical course of brucellosis and to estimate case-fatality rates. Additionally, reporting of methodological choices should be improved to enhance transparency and comparability of estimates. These steps will increase the value of these estimates for policy makers.

## Author summary

Brucellosis is a bacterial disease transmitted to humans by consumption of contaminated, unpasteurized milk or through direct contact with infected animals and their excretions. This disease causes production losses and has major economic impacts on individuals and communities. The disability-adjusted life year (DALY) is a metric for measuring the burden. It summarizes mortality (years of life lost) and morbidity (years lived with disability) into a single metric. This review aimed to identify existing brucellosis burden of disease studies and analyse their methodological choices, assumptions, and uncertainties. The results suggested that some parameters carry considerable uncertainty, particularly mortality and disease duration. This highlights the importance of strengthening routine reporting systems, collecting better mortality data and conducting further research on the course of brucellosis. Additionally, estimates of DALYs will benefit from a deeper understanding of the symptoms and the different sources of attribution. Finally, current reporting of methodological choices should be improved to enhance transparency, comparability, and consistency of brucellosis burden.

## Introduction

Brucellosis is globally one of the most widespread zoonoses and is caused by the genus *Brucella* [1]. This bacterial disease can be transmitted from animal reservoirs, such as cattle, sheep, goats, and pigs, to humans, through consumption of raw animal products, most commonly unpasteurized milk and soft cheese. It can also be transmitted through direct contact with infected animals and animal health workers [2–4]. Brucellosis has relatively non-specific symptoms, making it difficult to diagnose and differentiate from diseases like malaria, typhoid, or tuberculosis [4,5]. However, specific complications may occur, such as endocarditis, epididymo-orchitis and meningoencephalitis [6]. In addition to its clinical impact, brucellosis also has significant economic ramifications due to losses in animal production and time lost from work by patients [6,7].

Brucellosis has been prioritized in animal health for decades, with examples of successful eradication campaigns in cattle in Europe, the Americas and other high income countries [8–10]. These programs have been shown to be successful technically and economically [8–10] yet there have been difficulties replicating these successes in other species. Despite the recognition of its importance by animal health systems, the high level of *Brucella* infections in many areas of the world in both human and animal hosts and the status as a neglected zoonosis by the World Health Organization (WHO), brucellosis is rarely prioritized by human health systems. [11,12]. To address this gap, researchers and public health institutes worldwide have initiated burden of disease assessments in humans based on the Disability-Adjusted Life Year (DALY), a summary measure of public health widely used to quantify and compare disease burden. DALYs integrate the effects of mortality (Years of Life Lost, YLL) and morbidity (Years Lived with Disability, YLD) into a single metric and allow for the incorporation of different health states, as defined by the disease model [13]. In spite of the role that DALYs and other health metrics have played in informing national and global priorities, brucellosis DALYs estimates remain globally incomplete and infrequently undertaken. For instance, according to the WHO Foodborne Disease Burden Epidemiology Reference Group (FERG), brucellosis caused 393,239 foodborne illnesses in 2010, resulting in 124,884 DALYs worldwide [14]. To put these results into context, FERG estimated that in 2010, a total of 33 (UI 25–46) million DALYs were caused by 31 key hazards investigated [15]. These findings do not include the burden of direct transmission between animals and humans, thus, they are likely underestimated. The aim of the Global Burden of Disease (GBD) study is to quantify health loss from hundreds of diseases, injuries, and risk factors. Currently, it does not include brucellosis as a standalone cause of death and disability in their studies [16].

DALY calculations entail several methodological decisions and assumptions, which may limit comparability and interpretation of results generated by different studies, thus jeopardizing their relevance to policymakers as a prioritisation tool. Other commonly used methods to estimate disease burden, such as mortality and incidence, do not embrace the complexity of a disease. Different burden of disease estimates not only vary in terms of the geographic scope and temporal range they are conducted for, but also in fundamental methodological decisions and assumptions involved in the underlying calculations. Understanding both is critical to appreciating differences between studies and estimates, and a necessary part of the ongoing improvement in estimation efforts. This review will inspect key methodological choices underpinning DALY calculations in brucellosis burden of disease assessment. For instance, YLDs may be calculated from an incidence or a prevalence approach. The former approach considers the future health impact due to current exposures (YLD = incidence * duration * disability weight), while the latter approach considers the current health impact due to past exposures and emphasizes its contemporary impact on health systems (YLD = prevalence * disability weight) [17,18]. In both perspectives, disability weights (DWs) are an essential component. They translate morbidity into healthy life years lost, thus enabling comparison of morbidity and mortality. A DW is anchored from 0 (perfect health) to one (death) and it can be interpreted as the proportional reduction of perfect health due to an adverse health state [19]. Another crucial choice is the life expectancy table used for calculating YLLs, which defines the weight given to age-specific deaths. DALY calculations may also apply social weighting to give a different value to healthy life years lost at different ages (i.e., age weighting [19,20]) or time periods (i.e., time discounting [13]). Age weighting reflects the perspective that a year of life lived at one age is worth more than another. Similarly, time discounting indicates that the benefits and costs of today are valued more than those in the future. A final methodological choice is the construction of the disease model, which defines the health states that will be considered in the estimates and the possible transition between states [17]. In addition to these intrinsic methodological choices, DALY calculations are often hindered by data gaps and uncertainties, and different methods exist to deal with these limitations.

Dean et al. conducted two systematic reviews on specific brucellosis outcomes, namely its prevalence and clinical manifestation [4,21]; however, no comprehensive review on burden assessments has been carried out yet. Thus, this systematic review aims to identify existing brucellosis burden of disease studies and to analyze their methodological choices, assumptions, and uncertainties. As a result of this assessment, knowledge gaps will be identified which need to be addressed to generate improved and comparable brucellosis burden of disease estimates.

The paper will focus on the human health burdens of brucellosis, it will not address the estimations of burdens in the livestock sector, which will be covered in other areas of the Global Burden of Animal Diseases programme (GBADs) [22]. Its contribution to GBADs is to improve understanding of the impact of animal diseases on society, including human health. Zoonotic diseases that cause health issues and burdens to people and the animals they keep are problematic in society, identifying where burdens occur will help policy makers in their decisions on resource allocation and legislation.

## Methods

### Data sources and search strategy

This systematic literature review followed the Preferred Reporting Items for Systematic Reviews and Meta-analyses (PRISMA) Statement (S1 PRISMA Checklist) [23]. The protocol of this review was not registered. However, a protocol was done before starting the review and was shared with GBADs to ensure internal consistency and validity (S1 Table). The databases PubMed, Embase and Web of Science were systematically searched. The search strategy was carried out on 23 July 2021, thus the review included all the literature published until week 28, 2021; The following terms were used: Brucella, brucellosis, disability-adjusted life year, years of life lost, years with disability, cost-effectiveness and cost-effectiveness analysis.

The search strategy, including Boolean operators, is presented in S1 Search Strategy. A grey literature search was also conducted using a selection of databases (i.e., OpenGrey, OAIster, and the Journals Online project (JOLs)). A further search within the reference lists of included publications was performed to identify eligible burden studies which were not flagged by the initial search.

### Eligibility criteria

Peer-reviewed articles and grey literature published between January 1990 and July 2021 were included. Studies published before 1990 were omitted since the DALY metric was introduced in the early 1990s [13]. All studies quantifying DALYs were included, whether the primary purpose was a burden of disease assessment or not (e.g., cost-effectiveness studies). Additionally, only studies focusing on brucellosis in humans were elegible for inclusion. No language restrictions were applied. If necessary, help with translation was asked from other members of GBADs. Lastly, due to the small literature set, no critieria was applied for the study type; however, conference proceedings, abstracts, letters to editors and general correspondence were excluded, since they would not have all the details needed for this review.

### Data screening, selection, and extraction

Rayyan.ai [24] was used to manage citations and detect duplicates. First, duplicate entries were identified by considering the authors, the year of publication, the title of the article and the volume, issue, and page numbers. When in doubt, the abstract texts were compared. Subsequently, the sources were screened for eligibility (title–first step; abstract–second step; full-text–third step). The screening process was conducted by one researcher (CDB); when in doubt, articles were discussed with the study supervisor (BD). Additionally, a sample of 10% of all the retrieved articles was separately screened by another researcher (CF) to ensure that the selection was based on the criteria mentioned above and not on personal decisions. No differences were noticed in the sample screened by the two researchers (CDB and CF).

Finally, a database of all retrieved publications that met the eligibility criteria was created. For each eligible paper, an extraction grid (S1 Extraction Grid and definition) was used to gather data regarding study information and characteristics, i.e., data sources for epidemiological data, data adjustment, methodological perspective used to calculate YLDs and YLLs, and the DALYs result. The systematic review of Haagsma et al. [20] was used as a blueprint when creating the data extraction grid; in particular, the same categories proposed by Haagsma et al. [20] were used when collecting incidence and prevalence data. Additionally, the data extraction sheet from Charalampous et al. [25] was adopted in order to better fit the aim of this review and the disease. The data extraction was piloted with the FERG study [14], and no revision of the extraction table was necessary for this review. A separate grid was created (S1 Extraction Grid and definition) to collect the disease model information, i.e., the included health states and their transition probabilities, disease weights and durations. Additionally, a narrative summary for each study was made, including limitations and reasoning for the methodological choices made by the authors. Data extraction was performed by CDB, while NV reviewed. Currently, there are no well-defined tools available to assess the quality of DALY estimation studies. Due to the aim of the review, we decided to include all the eligible studies to have a complete vision of the current methodological choices and assumptions that have been used. This assessment can form the basis for a standardized tool or checklist to report and appraise methodological choices and uncertainties when estimating DALYs.

The display of the results has been done by using R program and Drawio. The shapefile for the map is part of rmìnaturalearth and rnaturalearthdata package in R program.

## Results

### Flow of selected studies

**Fig 1:**
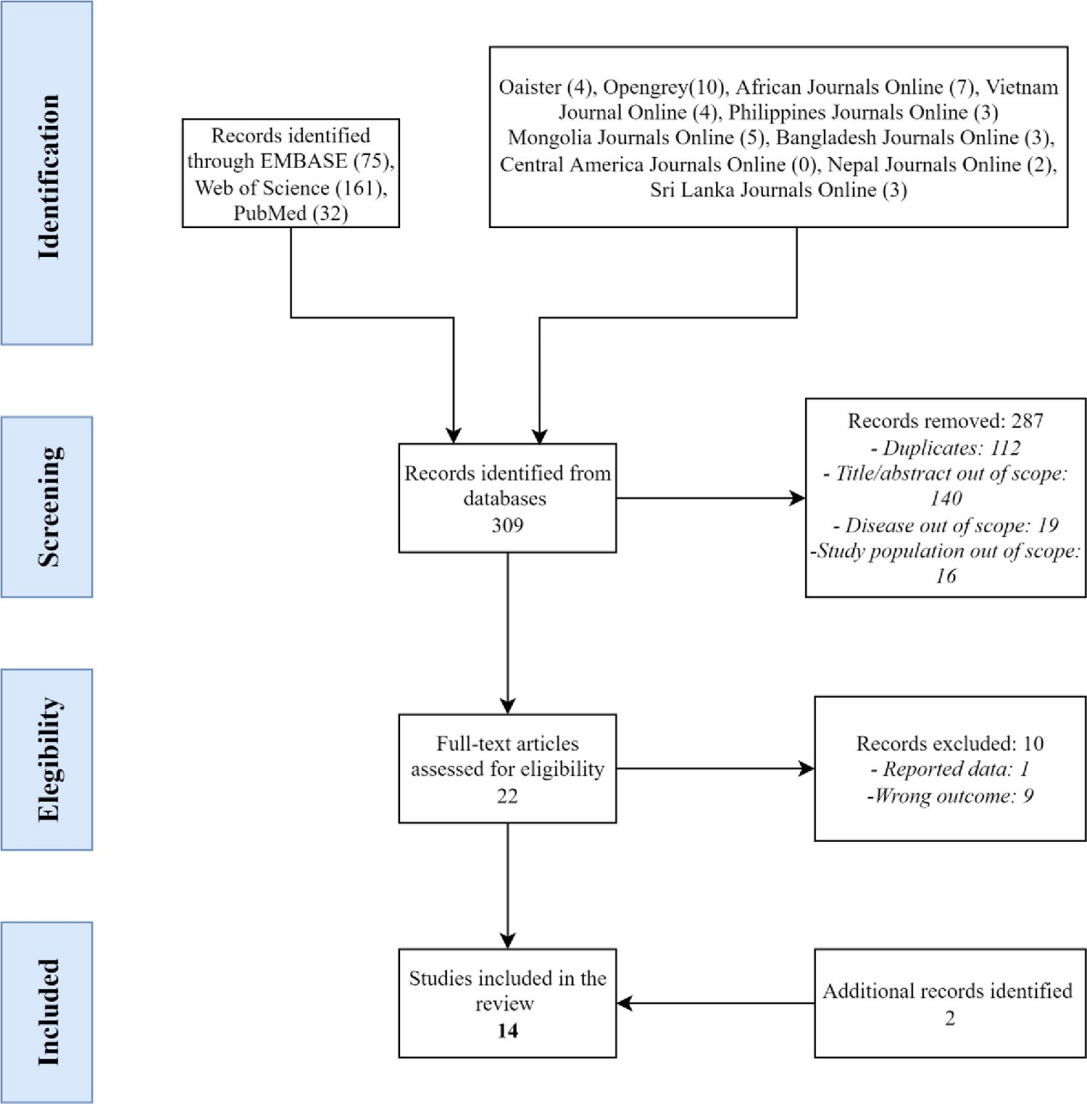
Flow chart of selected studies.

Fig 1 shows a detailed flow diagram for selecting articles included in the systematic review. In total, 309 articles were retrieved. After the removal of duplicates and the screening for eligibility, 22 studies were retained for full-text screening. Two additional suitable studies were found through reviewing the list of references. In the end, fourteen studies were included for data extraction.

### Number of burden of disease studies per location and year of publication

**Fig 2:**
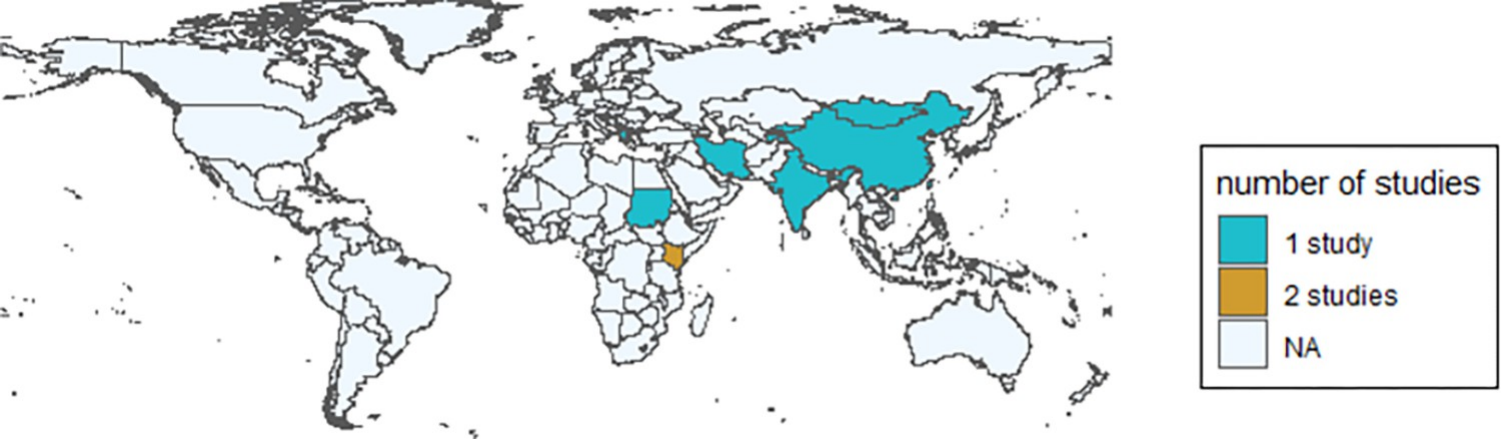
Countries with national or subnational Brucella burden studies estimates. Mapping and statistical testing were performed in R version 4.2.1. Maps were generated using the following packages: ggplot, rnaturalearth, rnaturalearthdata and sf.

Out of the fourteen eligible articles, only one estimated the brucellosis burden of disease globally, the FERG study, by Kirk et al. [14]. Others were national or subnational studies, set in Iran (2) [26,27], Kenya (2) [28,29], Greece [30], India [31], Kazakhstan [32], Kyrgyzstan [33], Mongolia [34], Sudan [35], China [36], Taiwan [37], and Tajikistan [38](1 each) (Fig 2). One of the studies set up in Kenya [29] and the study in Sudan [35] were sub-national studies, where they presented only sub-national estimates.

**Fig 3:**
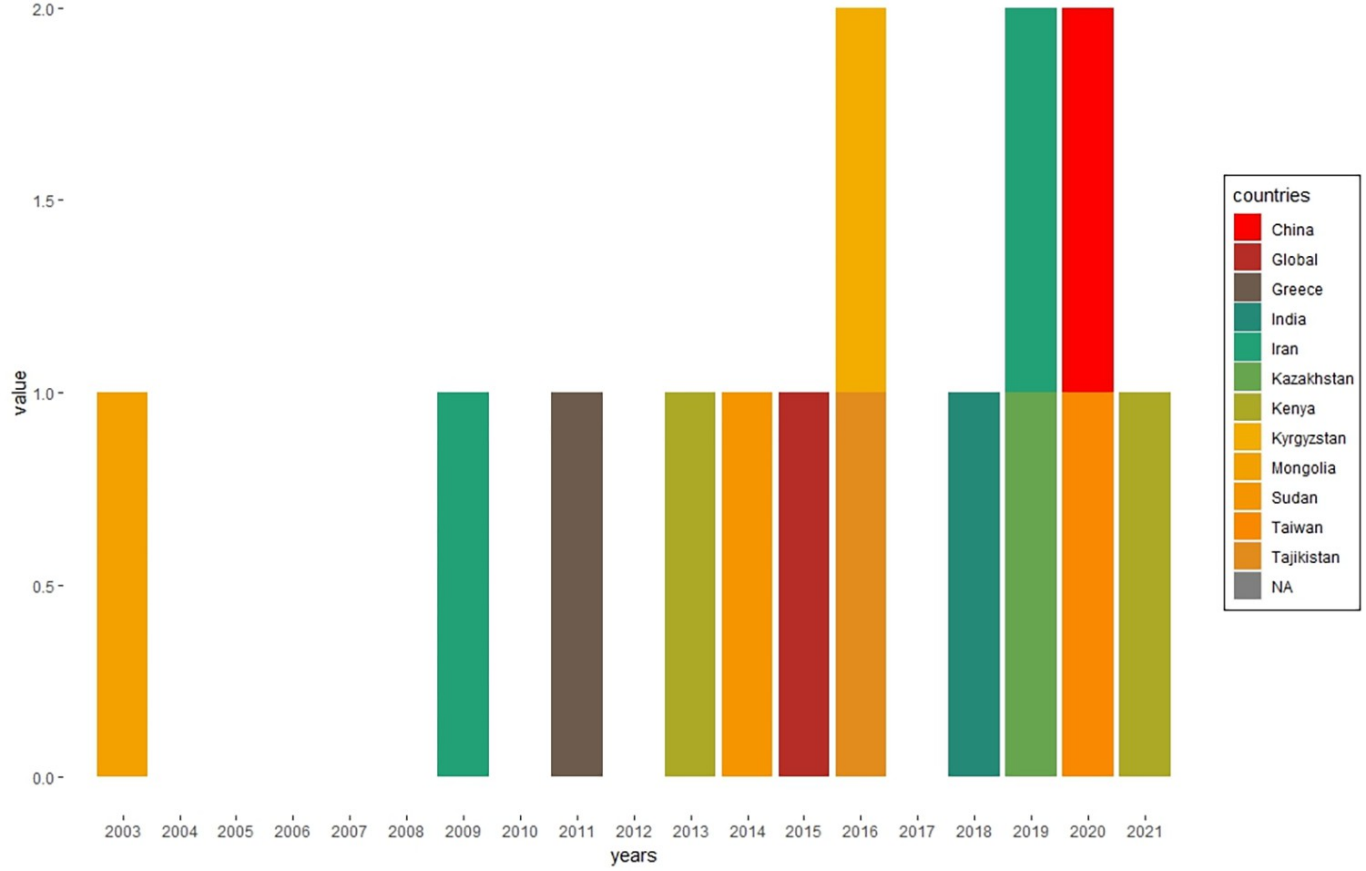
Existing *Brucella* burden of disease studies by year of publication.

Regarding the publication years, the first burden study dates back to 2003, while the most recent one was published in 2021. Fig 3 depicts that over the 2003–2021 period the number of published brucellosis burden of diseases study consistently ranged between 0 and 2.

### Epidemiological data

The selected studies used different epidemiological inputs as a source for DALYs calculation. Most of the studies, eleven out of fourteen, retrieved incidence data from laboratory-confirmed cases [7,26,28–33,35–37]. In addition, three studies collected the information through previous literature [14,31,33] and one study used data from syndromic surveillance [27]. Lastly, one study [31] obtained the incidence by dividing the number of seropositive cases by the duration of seropositivity (estimated to be 10.9 years). Notably, studies from Counotte et al. [33] and Singh et al. [31] sourced data from multiple inputs, such as routine data and surveillance systems, and previous literature. The former employed different sources to validate the findings, while the latter analyzed particular subgroups [31,33]. Eight studies assessed mortality. Five studies sourced the mortality indirectly using case fatality rates based on previous literature [7,14,30,32,33]; for example, Charypkhan [32] used the case fatality reported by a German study, which used a German data set [39]. The remainder derived mortality data directly from the national surveillance system as in the case of Piroozi et al. [27], or death registry as in the case of Navaghi et al. [26], Peng et al. [36] and Lai et al.[37].

### Data adjustments

Four studies considered and addressed the underestimation of incidence using two different approaches. Three studies used a data-driven approach. The study conducted in Kyrgyzstan [33] used prevalence data to address the underestimation in the official data. The study of Kirk et al. [14] computed a multiplier by comparing human incidence data from the World Organization for Animal Health (OIE) on countries “free of brucellosis in livestock” and the literature review by Dean et al. [21]. In contrast, Gkogka et al. [30] computed a multiplier from previous literature [40,41]. The other method to address underestimation was based on expert opinion. The value of the multiplier in the study by Piroozi et al. [27] was established by an expert panel. Interestingly, Brink conducted a scenario analysis with different underestimation rates, but the main results presented in the study did not consider underestimation [28].

Three studies assessed the possibility of missing values among model parameters and handled them in various ways. Specifically, Counotte et al. [33] used data from neighboring countries or overlapping regions for missing prevalence data; Kirk et al. [14] dealt with missing incidence data by computing the Bayesian log-normal random effect. Finally, Singh et al. carried out data triangulation to estimate the missing value of specific subgroups, such as the number of para-veterinarians [31].

### Methodological choices

#### General disability-adjusted life years methodology

Table 1 shows in details the different component of DALY, extracted from the articles. Not all studies considered YLLs caused by brucellosis. In fact, five studies explicitly stated that they did not consider them [26,28,29,31,35], while one study did not provide any information (Table 1) [38]. In the study of Naghavi et al. [26], the official registry did not report mortality data for brucellosis. Brink [28], Singh et al.[31] and Alumasa et al. [29] explained their decision on the basis of previous studies and national surveys that did not report brucellosis mortality data. Alternatively, Elkhansaa and Angara did not estimate YLLs from its perspective that “brucellosis is not a fatal disease” [35]. The remaining studies (N = 7) employed life expectancy tables from different sources to calculate YLLs (Table 1). Three studies [14,32,33] used WHO 2050 [42]; three studies used country-specific life tables [27,30,37]; whilst two study did not specify the life table chosen [7,36]. Finally, all the studies (N = 14) followed an incidence-based approach to calculate YLDs. None adjusted for comorbidity.

**Table 1:** Summary of disability-adjusted life year calculation methods for brucellosis.

Region	Study	General		YLL	YLD				Results	
		Discount rate	Age weighting	Life table	Approach	Duration source	Disability weights source	Archetypes used in the disease model	Estimated llnesses	DALYs per CASE
Global	Kirk et al., 2015 [14]	No	No	WHO 2050	Incidence	Literature (Dean et al., 2012) [4]	GBD 2010 [43]	Disease model 3	832,633	0.3
National	Counotte et al., 2016 [33]	No	No	WHO 2050	Incidence	Literature (Kirk et al., 2015) [14]	GBD 2010 [43]	Disease model 3	2,435	0.29
	Gkogka et al., 2011 [30]	No	No	Country-specific	Incidence	Literature (Roth et al., 2003)	GBD 1990 [44]	Disease model 2	64	0.62
	Roth et al., 2003 [7]	Yes	Yes	Not stated	Incidence	Literature (Beklemishew, 1964) [45]	GBD 1990 [44]	Disease model 2	1,482	0.62
	Piroozi et al., 2019 *(for incidence data S3)* [27]	Yes	No	Country-specific	Incidence	Expert	Burden of disease and injury in Iran 2003 [26]	Disease model 2		0.17
	Singh et al., 2018 [31]	No	No	-	Incidence	Literature (Roth et al., 2003) [7]	GBD 1990 [44]	Disease model 1	-	0.90
	Charypkhan et al., 2019 [32]	No	No	WHO 2050	Incidence	Literature (Bosilkovski et al., 2010)	Dean et al. (chronic brucellosis) [4]	Disease model 2	1,334	0.53
	Kurbonov et al., 2016 [38]	Not stated	Not stated	-	Incidence	-	-	-	-	1.69
	Alumasa et al., 2021 [29]	No	No	-	Incidence	Literature (Roth et al., 2003) [7]	Dean et al. (acute brucellosis) [4]	Disease model 1	-	0.68
	Elkhansaa and Angara 2014 [35]	Yes	Yes	-	Incidence	Not stated	GBD 1990 [44]	Disease model 1	176	1.00
	Peng et al., 2020 [36]	Yes	No	-	Incidence	Literature (Dean et al., 2012) [4]	Literature (Dean et al., 2012) [4]	Disease model 3 (No orchitis)	-	0.13
	Brink, 2013 [28]	Yes	No	-	Incidence	Literature (Kunda et al., 2007)	Dean et al. (acute brucellosis) [4]	Disease model 1	75,256	0.10
	Lai et al., 2020 [37]	No	No	Country-specific	Incidence	Literature (Kirk et al., 2015) [14]	WHO/FERG [14]	Disease model 2	-	-
	Naghavi et al., 2009	Yes	Yes	-	Incidence	Expert	Expert panel	Disease model 1	-	-

Five out of the fourteen studies applied age-weighting or time discounting rates. In particular, three studies applied both age-weighting and time discounting [7,26,35] (Table 1). Elkhansaa and Angara [35] and Naghavi et al. [26] chose, respectively, a rate of 0.04 and 0.3. In Roth et al.’s study, different time discounting rates (3% and 5%) were applied, with an age-weighting of 0.16 [7]. Piroozi et al. [27], Peng et al. [36] and Brink [28] only considered time discounted at 3%.

### Disease model choices

#### Health states

**Fig 4:**
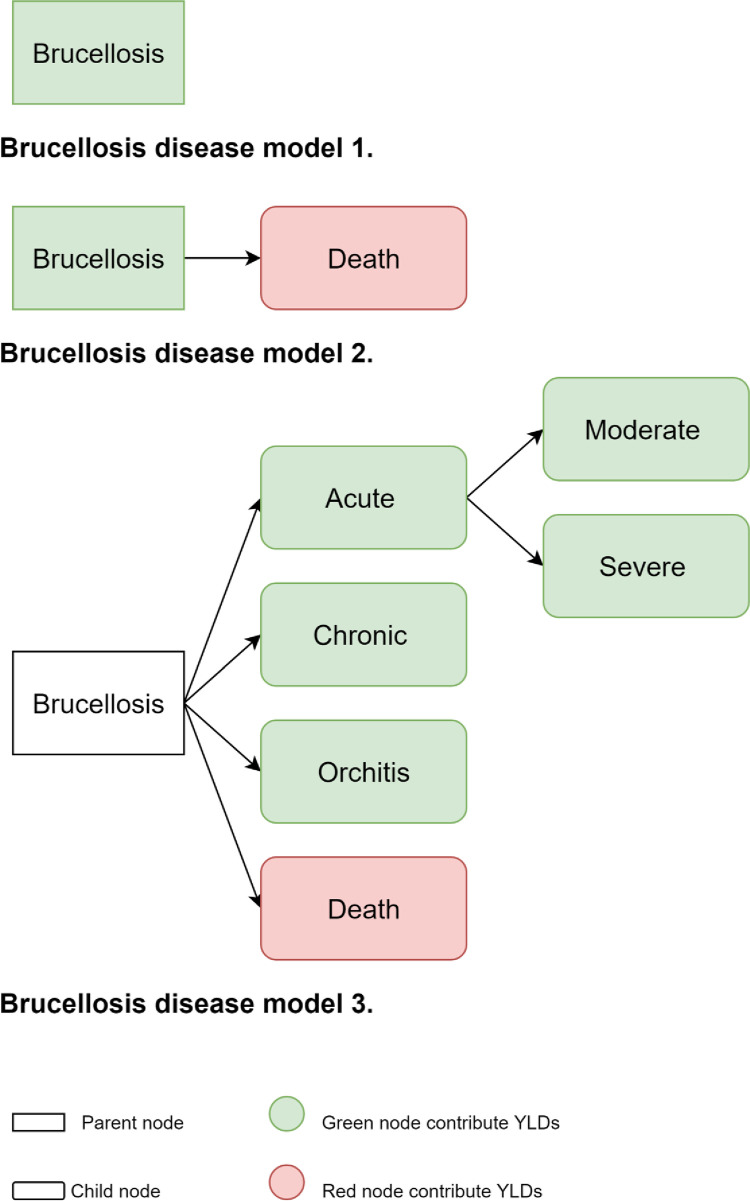
Disease models for brucellosis. Rectangles define the parent nodes, while rounded rectangles define the child nodes. White nodes do not contribute directly to the DALY estimate; green nodes contribute YLDs; and red nodes contribute YLLs. These models are not based on the biological or clinical pathway but they are computational disease models.

Out of the fourteen studies that calculated DALYs for brucellosis, twelve provided information regarding the disease model, including the different health states. All the studies applied one of the three archetypes, which are outlined in Fig 4. Kirk et al. [14] and Counotte [33] used the same disease model (Fig 4, Brucellosis disease model 3), developed by Kirk et al. [14], entailing six different health states (acute, severe, moderate, chronic, orchitis and death). Furthermore, they defined the probability of developing each health state. Kirk et al. [14] reported the different GBD proxy health states used to determine the disability weights that they implemented. Additional details can be found in the extraction grid (S1 Extraction Grid and definitions). Although Counotte [33] employed Kirk’s health model, some differences are identifiable, especially in the duration of chronic brucellosis, but no explanation is provided for these differences. Five studies presented two health states (a general “brucellosis” and death) (Fig 4, Brucellosis disease model 2). Peng et al. [36] applied disease model 3; However, he did not provided the probability of developing orchitis. The rest of the studies (N = 5) reported only brucellosis as a health state (Fig 4, Brucellosis disease model 1). They did not report death as a health state, because they did not calculate YLLs, or did not consider brucellosis as a fatal disease.

#### Duration

The burden assessments retrieved the disease duration from different sources (Table 1). Whilst twelve studies reported how they obtained it, two studies did not specify the process [26, 38]. Ten nine derived the duration of brucellosis from the literature. Among those, three studies [29–31] retrieved this parameter from Roth et al. [7]. Roth et al. initially assumed 4.5 years, based on Beklemishew [45], but for the nature of the study, the median of the cumulated discounted DALYs was assumed, corresponding to 3.11 years. For this reason, discrepancies could be found in the studies that retrieved duration from Roth et al. [7]. While Gkogka et al. used the value of 3.11 years, Singh et al. and Alumasa et al. kept 4.5 years as the duration of brucellosis. Kirk et al. used the result provided by the literature review conducted by Dean et al. [4] and differentiated between acute infections (14 days) and chronic infections (6 months). Counotte et al. [33] and Peng et al. [36] employed the data from Kirk et al. as the source for the disease duration of brucellosis [14]. The burden of disease estimated by Lai et al. [37] also cited the duration presented by Kirk et al. [14], however, they did not regard the different health states separately but instead assumed an overall disease duration of 0.038 years (two weeks). Charypkhan [32] employed the duration from Bosilovski et al. [46] (0.21 years or ten weeks) and confirmed it with the data reported by Dean and colleagues [21]. Brink derived it (0.5 years or six months) from studies by Kunda et al. [47], Kursun et al.[48] and Muriuki et al. [49] (S1 Extraction Grid).

Two studies, both set in Iran, used expert opinions to estimate the duration of brucellosis. Piroozi et al. described that they held an expert panel with infectious diseases specialists [27]. The duration has been assumed to be 0.75 years. For *The burden of disease and Injury in Iran 2003*, the duration was estimated with expert input from Iranian disease epidemiologists and clinical specialists [26]. The resulting disease duration was not reported. The study by Elkhansaa and Angara assumed three years for the duration of illness; however, it failed to report the source [35]. More information can be found in Table 1 and in S1 Extraction Grid.

#### Disability weights

Another sensitive methodological choice that needs to be considered is the disability weight (DW). Out of fourteen studies, twelve reported the source for their DW (Table 1). It varied from 0.053 [14] to 0.23 [26,27]. Two studies [14,33] derived the DW from proxy health indicators in the GBD 2010 [43]. For example, their DW for acute and severe brucellosis corresponded to the GBD 2010 definition of “infectious disease: acute episode, severe” (S1 Extraction Grid and definitions). Lai et al. [37] retrieved the DW (0.092) from Kirk et al., [14] and have subsequently calculated the weighted average between acute episode severe (DW = 0.133) and acute episode moderate (DW = 0.051). Alternatively, four studies [7,30,31,35] used DWs from GBD 1990 [13]. Studies that referred to the GBD 1990 have a DW of 0.2, also defined as a disability of class II. A DW of 0.2 according to the GBD 1990 correspond to a condition that is “painful and affecting occupational ability even during period of remission” [13]. Furthermore, four studies retrieved the DW from Dean et al. [4]. Dean et al.[4] proposed two different DWs, based on GBD 2004: 0.15 for chronic brucellosis and 0.19 for acute brucellosis. On the one hand, Charypkhan et al. [32] utilized 0.15, which is the DW proposed by Dean et al. for chronic brucellosis [4]. On the other hand, Brink [28] took the acute state (0.19) as reference. Alumasa et al. [29] also refer to Dean et al. [4] as the source for deriving their DW of 0.18. Piroozi et al. (0.23) sourced the DW from the *Burden of disease and injury in Iran in 2003 using the Delphi method* [26]. A summary can be found in Table 1. There is great variation in the value of DALY per case; the minimum DALY per case is 0.10 presented by Brink [28], followed by the value of Piroozi et al. [27] (0.17). On the other end of the spectrum, there are the estimates presented by Kurbonov et al. [38] and Elkhansaa and Angara [35], respectively 1.69 and 1.00.

#### Source attribution

Four studies explored and took into account different routes of transmission for brucellosis (S1 Extraction Grid). Three studies considered transmission via food as a transmission route. They retrieved the percentage attributable to foodborne exposure from previous literature [30,37] or from global expert elicitation [14]. In contrast, the forth study [31] considered occupation as a transmission route. Information was retrieved from data regarding the prevalence of human brucellosis among veterinary personnel, abattoir workers and livestock farmers from the published scientific literature. More information on the attribution source can be found on S1 Extraction Grid and definition.

### Uncertainty analyses: Analyzing model specification, parameters and uncertainty

Four studies conducted their own scenario analysis. Different elements were used when doing a scenario analysis: different life expectancy tables (country-based and West level 26) [30], discount rates (0.3 and 0.5) [7], with or without age weighting and time discounting [31], and different degrees of underestimation (0, 5, 20, 50, 75, 90%) [28]. Uncertainty was modelled by five studies using Monte Carlo simulations [7,14,30,31,33].

## Discussion

This review provides a comprehensive overview of the studies that calculated the burden for human brucellosis that were published from January 1990 to July 2021. The aim was to identify existing brucellosis burden of disease studies, and analyze their methodological choices, assumptions, and uncertainties. In total, fourteen studies met the inclusion criteria. The OIE World Animal Health Information System database collects information on animal health around the world. According to the OIE data from 2005 to 2022, *Brucella* is reported in animals in the Mediterranean region, in the Middle East, Russia, Sub-Saharan Africa, in some countries of Latin America and in central and south Asia [50]. Due to brucellosis’ mode of transmission, it can be assumed that it affects humans in the same locations or in people who travel to these areas. Despite repeated documentation of brucellosis within animal hosts, there is no human burden data available in some of the locations that are affected. Indeed, most of the studies included in this review were from Eastern Europe, Asia, and Sub-Saharan Africa. No studies were identified from North, Central and South America or South-East Asia. This is in line with the observations by Dean et al., who noted in their reviews [4,21] that the same geographical areas were missing brucellosis burden data. This could potentially reflect either a lower disease presence or poorer brucellosis surveillance. Thus, the (national) studies that we found present only a limited picture of the burden of brucellosis.

Our review revealed large methodological variations in available burden of disease estimates for brucellosis. Indeed, differences are visible when scrutinizing the DALYs per case presented by the articles (Table 1). The variations in the DALYs per case highlight the impact of the methodological choices and assumptions proposed by the authors (Table 1). For instance, Brink et al. [28] have the lowest DALYs per case (0.10); this is due to methodological choices, such as not accounting for mortality, applying time discounting, and having chosen a shorter duration (6 months), and a DW of 0.19. In contrast, the study conducted by Gkogka et al. [30] presented different DALYs per case based on different methodological choices. Gkogka et al. accounted for YLL, with a case fatality of 2%. Time discounting and age-weighting were not applied. Additionally, the chosen duration and DW were 3.11 years and 0.2, respectively. These two examples illustrate the considerable direct impact of the methodological choices on the estimates. This implies that comparisons of available (national) estimates with other countries should only be done with caution. A method to enhance direct comparison between the different DALYs estimates would be to recalculate the DALYs using a single consistent methodology or by choosing one DALYs per case presented by one of the studies and applying it across the studies.

Generally, when conducting burden of disease studies is important to report the reference population (e.g., whole country versus a specific region), since the population of reference is important when calculating the DALY. Additionally, it is importanto to provide rates per 100.000 or DALY per case. Most of the study provided the reference population, thus where do not provided we estimated the rate per 100.000.

In what follows, we describe the main implications of the methodological differences observed. All the studies employed an incidence-based approach. The incidence-based approach reflects the future burden based on current events, where all health outcomes are assigned to the initial event [20]. For the burden of brucellosis, the incidence-based approach is often mentioned to be preferable because it is sensitive to current epidemiological trends and it is more consistent with YLLs, which by definition follow an incidence approach (mortality can be seen as the incidence of death) [17,18]. Additionally, laboratory-confirmed cases were often used to compute the incidence. However, reliance on solely laboratory-confirmed cases would result in underestimation [20]. A correction for potential underestimation was only included in four studies. Underestimation of the brucellosis burden of disease has implications in priorities settings and could undermine the value of the studies as advocacy tool.

The majority of mortality data were obtained indirectly. Moreover, some studies stated that the official registries reported no data on mortality. This could happen due to a lack of data, indicating that fatal outcomes of brucellosis are rare, or due to inadequate cause of death reporting systems. Calculating burden of disease estimates requires high-quality mortality data. The absence of those has an important consequence in the calculation of YLLs, although in the case of brucellosis, most of the burden relies on YLDs. Nevertheless, the omission of YLLs will cause an underestimation of the burden.

Differences in the duration values were observed, which revealed that there is uncertainty surrounding the length of this disease. The duration utilized by the articles varied from two weeks to 4.5 years. Counterintuitively, the duration did not have a connection with the archetype (see Fig 4) that has been used. For example, the duration of 4 or 4.5 years was used for the estimates of Singh et al. and Roth et al. and they applied the first and second archetype of brucellosis’ disease model respectively. Meanwhile Kirk et al. and Counette et al. applied archetype 3 but they used shorter durations also for the chronic phase (six months).

All studies took the duration data from previous literature or expert opinion, and no study used duration that was directly data-driven. In particular, studies that retrieved it from previous literature used the same few sources, which could probably be context-specific and/or outdated. For instance, as in the case of Beklemishew [45], used by Roth et al., the study was based on the clinical data of 1000 untreated patients in the Russian Federation. This raises questions on the validity of the selected values in contemporary contexts. Another finding relating to the disease model was that most studies did not consider different health states and symptoms. This could be caused not only by the lack of clinical data on brucellosis sequelae but also by the lack of knowledge on the disease course. Additionally, none of the studies referred to abortions in pregnant women as a health state, even though different studies provide evidences of the adverse effects of brucellosis on pregnancy outcome [51–53]. Few studies took into account the routes of transmission of brucellosis and the relative importance of these routes. A deeper understanding of the transmission could help gain a better familiarity with sources of attribution and help develop effective prevention measures. Finally, we observed that not many disease burden studies assessed uncertainty. Ideally, studies should clearly describe the uncertainties in their data and methods, and quantify them. Indeed, this will strengthen the validity of the results and allow better comparability across different studies.

### Limitations

The review has some limitations. First, ongoing estimates or studies that were performed but not documented in peer-reviewed articles or grey literature could potentially be missing. To limit this possibility, reference lists were checked. Second, the search strategy was only conducted in English. This could potentially lead to missing out on some eligible and valid studies in other languages. Additionally, the screening and data extraction process was performed only by one researcher (CDB). To limit the selection bias, another reviewer screened a randomly selected sample and and the data extraction was also revised by a second researcher.

Finally, there is no standardized way of evaluating DALYs calculations or disease models currently in place. Thus for this review, we had to come up with an approach ourselves, which was inspired by previous literature along with the work done by the European Burden of Disease Network [25].

### Future prospects

This review aimed to report different methodological choices that have been made in assessing the burden of brucellosis in humans. The findings of the review can serve as a base to improve future burden of disease estimates, especially global analyses such as FERG and GBD. The OIE data [50] showed that brucellosis is spread worldwide; nevertheless, human burden of disease estimates are only available for a limited number of countries where animal brucellosis is reported to occur. Better synergies between animal and human data for brucellosis will not only provide better estimates but also a clearer view on transmission and on effective interventions. Indeed, this review will support the GBADs programme to develop a systematic methodology to describe the impact of animal diseases on society, including human health, and to close the gap between human and animal health.

From the review, it appears that incidence data, and especially mortality and duration, should be better addressed with more data-driven approaches. Consequently, further research needs to be done for these parameters across multiple countries. Better routine reporting, new clinical studies focusing on duration and incidence, and better mortality registries would help fill these gaps. Additionally, more evidence is needed on the link of specific brucellosis symptoms with health states to better understand the course of the disease. Finally, exploring sources of attribution will give a better understanding of the transmission and prevention of brucellosis.

This review found a great variety in the methodological choices and assumptions used to calculate DALYs and numerous inconsistencies in reporting methods and assumptions. Thus, there is a need for a more standardized reporting system for DALYs estimates, which could resemble a checklist that reports the methodological choices and assumptions. The adoption of this tool will enhance the transparency and understanding of the methodological choices and the subsequent reuse of these estimates for prioritisation purposes.

## Conclusion

This review aimed to explore the different methodological choices and assumptions used to quantify the DALYs for brucellosis. The results suggested that some parameters carry considerable uncertainty, particularly mortality and disease duration. This highlights the importance of strengthening routine reporting, collecting better mortality data and conducting further research on the course of brucellosis. Additionally, estimates of DALYs will benefit from a deeper understanding of the symptoms of the disease and the different sources of attribution. Current reporting of methodological choices should be improved to enhance transparency, comparability, and consistency of brucellosis burden of disease estimates. Finally, within the scope of the GBADs programme, this review supports the development of a systematic methodology to describe the impact of animal diseases on society, including human health, and to strengthen the connection between human and animal health.

## Declarations

This research is on behalf of the Global Burden of Animal Diseases (GBADs) programme which is led by the University of Liverpool and the World Organisation for Animal Health (WOAH, founded as OIE) (https://animalhealthmetrics.org/). A full list of the GBADs collaborators can be accessed here: https://animalhealthmetrics.org/acknowledgments.

## Supporting information

S1 PRISMA checklist PRISMA Checklist. (PDF)

S1 Table Inception table. (DOCX)

S1 Search strategy Search strategy. (PDF)

S1 Extraction grid Extraction Grid. (XLSX)
