# Supplementary material for: Methodological choices in brucellosis burden of disease assessments: A systematic review
Source: PLoS Negl Trop Dis. 2022 Dec 13;16(12):e0010468. doi: 10.1371/journal.pntd.0010468 (PMC9794075; doi:10.1371/journal.pntd.0010468)
Supplement: S1 Table — (DOCX) [file pntd.0010468.s002.docx]

GBADs literature reviewing inception table

Note: Non-italicized elements are ‘essential’ and should be reported in the main review report or as supplementary material. Elements written in italics are ‘additional’, providing supplementary information or detail. This table is an adaptation of the [PRISMA 2020 checklist](https://www.bmj.com/content/372/bmj.n71/related#datasupp) [1].

| Section and Topic | Item  # | Elements recommended for reporting | Specific review details |
| --- | --- | --- | --- |
| TITLE |  |  |  |
| TITLE | 1 | - Identify the report as a systematic review in the title. - Report an informative title that succinctly provides key information about the main objective or question the review addresses including stating items such as: - The outcome domain(s) (e.g. epidemiological/animal husbandry measures/economic/socio-economic/specific research or policy method) and specific measure(s) (e.g. prevalence/fertility/cost of medicines) to be reviewed (outcomes), - The disease/condition of interest which the outcome measure is describing, - The population of interest (participants), - The geographical setting, - The time-period being examined.   i.e. Identifying [outcome measure and domain, if necessary] in [disease/condition of interest] in [population/time-period/spatial area]. e.g. “**Identifying the prevalence of foot and mouth disease in cattle in Ethiopia between 2010 and 2019**”.   - *Consider providing additional information in the title, such as the method of analysis used, the designs of included studies, or an indication that the review is an update of an existing review, or a continually updated (“living”) systematic review.* | Review on the brucellosis burden papers, with a focus on methodology |
| ABSTRACT |  |  |  |
| ABSTRACT | 2 | •  Report an abstract addressing each relevant item in the [PRISMA 2020 for Abstracts checklist](https://www.bmj.com/content/372/bmj.n160) (see box 2 within this) [1]. |  |
| INTRODUCTION |  |  |  |
| RATIONALE | 3 | Describe the rationale for the review in the context of existing knowledge - this should help readers understand why the review was conducted and what the review might add to existing knowledge.  **Essential elements**   - Describe the current state of knowledge and its uncertainties. - Articulate why it is important to do the review. - If other reviews addressing the same (or a largely similar) question are available, explain why the current review was considered necessary (for example, previous reviews are out of date or have discordant results; new review methods are available to address the review question; existing reviews are methodologically flawed; or the current review was commissioned to inform a guideline or policy for a particular organisation). If the review is an update or replication of a particular systematic review, indicate this and cite the previous review. | Brucellosis is one of the most widespread zoonoses in the world caused by the genus Brucella. This bacterial disease can be transmitted from animal reservoirs, such as cattle, sheep, goats, and pigs to humans, through consumption of unpasteurized dairy products and uncooked meat or direct contact with infected animals. Brucellosis does not only cause debilitating and disabling illness, but it also has major economic ramifications due to losses in animal production and time lost by patients.  Despite the high level of infection in many areas of the world, Brucellosis is rarely prioritized by health systems and is considered a neglected zoonosis by the World Health Organization.  Nevertheless, in 2006, the WHO launched an initiative to estimate the global burden for Foodborne disease, which included Brucellosis. Overall, Brucellosis was estimated to cause 393,239 (UI 143,815 – 9,099,394) illnesses in 2010 and resulted in 124,884 (43,153 – 2,910,416) DALYs.  It is important to note that there are different disease models, data sources and methodologies for calculating the BoD for Brucellosis. For this reason, this review aims to identify high quality burden of disease studies and to review the state of the art to understand better of the works that have been already done and the methodologies used. |
| OBJECTIVES | 4 | - Provide an explicit statement of all objective(s) or question(s) the review addresses, expressed in terms of a relevant question formulation framework. - It is useful to use a question formulation framework such as PEO (Population and their problems; Exposure; Outcomes or Themes. | **Objectives**:   This literature review aims to identify the available BoD studies for Brucellosis in order to review the current state of the arts and to explore the different methodological choices done in the BoD’s estimation.  The following research questions will be answered:   - How was the Burden of Brucellosis estimated? - Which data sources are used? What are the sources of epidemiological and clinical data? - Which disease models have been used? What sequelae have been included? |
| METHODS |  |  |  |
| ELIGIBILITY CRITERIA | 5 | - Specify all study characteristics used to decide whether a study is eligible for inclusion in the review. Consider the elements you wish to limit within search phrases, such as eligible study design(s) and setting(s) e.g. - How is the disease/condition of interest/research or policy method defined? If necessary, split into sub-concepts - Who should make the diagnosis? e.g. veterinarian, para-vet, livestock keeper - What are the most important characteristics that describe the study participants? - Are there any relevant demographic factors e.g. age, breed? - What is the setting? e.g. dairy system - How data-rich is the topic? – If data poor, then may need to review over a longer time-period - How will studies involving only a subset of relevant participants be handled? - Specify eligibility criteria with regard to report characteristics, such as year of dissemination, language, and report status e.g. whether reports, such as unpublished manuscripts and conference abstracts, are eligible for inclusion. *NOTE: you need to be able to clearly understand and report if studies were ineligible because the outcomes of interest were not measured, or ineligible because the results for the outcome of interest were not reported. Reporting that studies were excluded because they had “no relevant outcome data” is ambiguous and should be avoided.* - *Consider providing rationales for any notable restrictions to study eligibility. For example, authors might explain that the review was restricted to studies published from 2000 onward because that was the year certain information was first available.* - Specify any groups to be used in the synthesis (e.g. outcome and population groups) and link these to the comparisons specified in the objectives (item #4). | **Inclusion criteria**:   - Burden of disease methodology and calculation using the DALY framework - The burden of disease should concern humans - The paper should concern Brucella/brucellosis   **Exclusion criteria**:   - No burden of disease outcome (DALYs) - The burden of disease concern animals |
| INFORMATION  SOURCES | 6 | - Specify the date when each source (e.g. database, register, website, organisation) is to be searched or consulted. - If bibliographic databases are to be searched, specify for each database its name (e.g. MEDLINE), the interface or platform through which the database is to be searched (e.g. Ovid, EBSCOhost), and the dates of coverage (where this information is provided). - If study registers, regulatory databases and other online repositories are to be searched, specify the name of each source and any date restrictions that will be applied. - If websites, search engines or other online sources are to be browsed or searched, specify the name and URL of each source. - If organisations or manufacturers are to be contacted to identify studies, specify the name of each source. - If individuals are to be contacted to identify studies, specify the types of individuals contacted (e.g. authors of studies included in the review or researchers with expertise in the area). - If reference lists are to be examined, specify the types of references to be examined (e.g. references cited in study reports included in the systematic review, or references cited in systematic review reports on the same or similar topic). - If cited or citing reference searches (also called backward and forward citation searching) is to be conducted, specify the bibliographic details of the reports to which citation searching is to be applied, the citation index or platform to be used (e.g. Web of Science), and the date the citation searching will be done for. - If journals or conference proceedings are to be consulted, specify of the names of each source, the dates covered and how they will be searched (e.g. handsearching or browsing online). | The following databases will be used to search for literature:   - Pubmed - Web of Science - Embase |
| SEARCH STRATEGY | 7 | - Provide the keyword and phrase search terms to be used within the search. Split these into those describing the five sections discussed in #1 the study title, including: - The outcome domain(s) and specific measure(s) to be reviewed, - The disease/condition of interest which the outcome measure is describing, - The population of interest (participants), - The geographical setting, - The time-period being examined. - Describe any limits applied to the search strategy (e.g. date or language) and justify these by linking back to the review’s eligibility criteria. - If natural language processing or text frequency analysis tools are to be used to identify or refine keywords, synonyms, or subject indexing terms to use in the search strategy, specify the tool(s) used. - If a tool is to be used to automatically translate search strings for one database to another, specify the tool to be used. - If the search strategy is to be validated - for example, by evaluating whether it could identify a set of clearly eligible studies - report the validation process used and specify how studies will be selected for inclusion in the validation set. - If the search strategy is to be peer reviewed, report the peer review process to be used, whether there is an aim to publish the review protocol (in which journal/section), and whether a specify any tool was used. - If the search strategy structure adopted is not based on a PEO-style approach, describe the final conceptual structure and any explorations that were undertaken to achieve it. | Keywords and phrases:   - **Outcome domain and measures:** - Burden of disease - Disability-adjusted life year - Disability-adjusted life years - DALY - DALYs - Years of life lost - Years lived with disability - YLL - YLD - Cost-effectiveness - **Disease/condition of interest:** - Brucella - Brucellosis - **Population:** - Humans - **Geographical setting:** - Global estimates, national estimates, sub-national estimates and regionals estimates - **Time-period:** - 1994 – to June 2021 |
| RELEVANT PUBLICATIONS | 8 | - Provide hyperlinks (to Open Access sources) to publications already known to be highly relevant to the topic | - [Economic and health burden of brucellosis in Kazakhstan](https://pubmed.ncbi.nlm.nih.gov/31090193/) [2] - [World Health Organization Estimates of the Global and Regional Disease Burden of 22 Foodborne Bacterial, Protozoal, and Viral Diseases, 2010: A Data Synthesis](https://pubmed.ncbi.nlm.nih.gov/26633831/) [3] |

**References**

1. Page MJ, Moher D, Bossuyt PM, Boutron I, Hoffmann TC, Mulrow CD, et al. PRISMA 2020 explanation and elaboration: updated guidance and exemplars for reporting systematic reviews. BMJ. 2021 Mar 29;372:n160.

2. Charypkhan D, Sultanov AA, Ivanov NikolayP, Baramova SA, Taitubayev MerekeK, Torgerson PR. Economic and health burden of brucellosis in Kazakhstan. Zoonoses Public Health. 2019;66(5):487–94.

3. Kirk MD, Pires SM, Black RE, Caipo M, Crump JA, Devleesschauwer B, et al. World Health Organization Estimates of the Global and Regional Disease Burden of 22 Foodborne Bacterial, Protozoal, and Viral Diseases, 2010: A Data Synthesis. PLoS Med. 2015;12(12):e1001921.
