# Supplementary material for: Methodological choices in brucellosis burden of disease assessments: A systematic review
Source: PLoS Negl Trop Dis. 2022 Dec 13;16(12):e0010468. doi: 10.1371/journal.pntd.0010468 (PMC9794075; doi:10.1371/journal.pntd.0010468)
Supplement: S1 Search strategy — (PDF) [file pntd.0010468.s003.pdf]

Contents

Embase 1
History Web of Science 1
Pubmed 3

The search was carried out on the 23 July 2021.

Embase

1	Brucella/	4376
2	brucellosis/	11075
3	disease burden/	26005
4	disability-adjusted life year/	2665
5	years of life lost.mp.	2326
6	years lived with disability.mp.	970
7	Disability-adjusted life years.mp. or disability-adjusted life year/	5181
8	cost-effectiveness.mp. or "cost effectiveness analysis"/	186962
9	1 or 2	13341
10	3 or 4 or 5 or 6 or 7 or 8	217460
11	9 and 10	75

History Web of Science

16
(#14) AND #13 and **Humans** (Search within all fields)
Edit
Add to Search
45

15
(#14) AND #13
Edit
Add to Search
161

14
((((((((#12) OR #11) OR #10) OR #9) OR #8) OR #7) OR #6) OR #5) OR #4) OR #3
Edit
Add to Search
259,510

13
(#1) OR #2
Edit
Add to Search
12,407

12
TS=(cost-effectiveness)
Edit
Add to Search
108,176

11

TS=(YLD)
Edit
Add to Search
431
10
TS=(YLL)
Edit
Add to Search
568
9
TS=(years lived with disability)
Edit
Add to Search
10,904
8
TS=(years of life lost)
Edit
Add to Search
11,601
7
TS=(DALYs)
Edit
Add to Search
2,463
6
TS=(DALY)
Edit
Add to Search
3,105
5
TS=(Disability-adjusted life years)
Edit
Add to Search
3,924
4
TS=(Disability-adjusted life year)
Edit
Add to Search
3,924
3
TS=(Burden of disease)
Edit
Add to Search
134,584
2
TS=(Brucella)
Edit
Add to Search
9,294
1
TS=(Brucellosis)
Edit
Add to Search
7,672

PubMed

Search number	Query
**15**	((((((((((Burden of disease[Title/Abstract]) OR (Disability-adjusted life year[Title/Abstract])) OR (Disability-adjusted life years[Title/Abstract])) OR (DALY[Title/Abstract])) OR (DALYs[Title/Abstract])) OR (years of life lost[Title/Abstract])) OR (years lived with disability[Title/Abstract])) OR (YLL[Title/Abstract])) OR (YLD[Title/Abstract])) OR (cost-effectiveness[Title/Abstract])) AND ((brucellosis[Title/Abstract]) OR (brucella[Title/Abstract]))
**14**	(((((((((Burden of disease[Title/Abstract]) OR (Disability-adjusted life year[Title/Abstract])) OR (Disability-adjusted life years[Title/Abstract])) OR (DALY[Title/Abstract])) OR (DALYs[Title/Abstract])) OR (years of life lost[Title/Abstract])) OR (years lived with disability[Title/Abstract])) OR (YLL[Title/Abstract])) OR (YLD[Title/Abstract])) OR (cost-effectiveness[Title/Abstract])
**13**	(brucellosis[Title/Abstract]) OR (brucella[Title/Abstract])
**12**	cost-effectiveness[Title/Abstract]
**11**	YLD[Title/Abstract]
**10**	YLL[Title/Abstract]
**9**	years lived with disability[Title/Abstract]
**8**	years of life lost[Title/Abstract]
**7**	DALYs[Title/Abstract]
**6**	DALY[Title/Abstract]
**5**	Disability-adjusted life years[Title/Abstract]
**4**	Disability-adjusted life year[Title/Abstract]
**3**	Burden of disease[Title/Abstract]
**2**	brucella[Title/Abstract]
**1**	brucellosis[Title/Abstract]
